# A new species of *Tachycines* Adelung, 1902 (Orthoptera, Rhaphidophoridae, Aemodogryllinae, Aemodogryllini) from karst caves in Guizhou, China

**DOI:** 10.3897/zookeys.937.49173

**Published:** 2020-06-01

**Authors:** Xulin Zhou, Weicheng Yang

**Affiliations:** 1 School of Life Sciences, Guizhou Normal University; Guiyang, Guizhou 550031, China Guizhou Normal University Guiyang China; 2 Institute of Karst Caves, Guizhou Normal University, Guiyang, Guizhou 550031, China Guizhou Normal University Guiyang China

**Keywords:** Orthoptera, Tachycines (Gymnaeta), Ziyun

## Abstract

Tachycines (Gymnaeta) trapezialis**sp. nov.** (梯形裸灶螽) is described with specimens collected from Diaosiyan and Sanjiaoshan caves in Ziyun County, Guizhou, China. The unique trapezoidal shape of the epiphallus in males and the subgenital plate in females, which separate the new taxon from its congeners, are illustrated.

## Introduction

During scientific expeditions to the proposed Ziyun Nature Reserve for Hume’s pheasant (*Syrmaticus humiae*) in Ziyun County, Guizhou, China, nymphs and adults of a new species were collected in Diaosiyan and Sanjiaoshan caves in Ziyun County, Guizhou, China, in June and October of 2019.

These specimens were found to belong to the subgenusGymnaeta Adelung, 1902 in the genus *Tachycines* Adelung, 1902 and Tachycines (Gymnaeta) trapezialis sp. nov. is described herein. Nine valid species of the subgenus have been recorded from Guizhou Province, i.e. Tachycines (Gymnaeta) ferecaecus (Gorochov, Rampini & Di Russo, 2006), Tachycines (Gymnaeta) proximus (Gorochov, Rampini & Di Russo, 2006), Tachycines (Gymnaeta) chenhui (Rampini & Di Russo, 2008), Tachycines (Gymnaeta) latellai (Rampini & Di Russo, 2008), Tachycines (Gymnaeta) zorzini (Rampini & Di Russo, 2008), Tachycines (Gymnaeta) solida (Gorochov, Rampini & Di Russo, 2006), Tachycines (Gymnaeta) borutzkyi (Gorochov, 1994), Tachycines (Gymnaeta) dispar (Qin, Liu & Li, 2019) and Tachycines (Gymnaeta) lalinus (Feng, Huang & Luo, 2019) (Gorochov, 1994; Gorochov et al. 2006; Rampini et al. 2008; Feng et al. 2019; Qin et al. 2019). Six of them have been found in caves of Guizhou (Gorochov et al. 2006; Rampini et al. 2008; Wen. 2018; Feng et al. 2019).

## Materials and methods

All specimens used in this study were preserved in 75% ethanol. Details of the morphology were studied under an Olympus SZ61 stereomicroscope. Male genitalia were preserved in mixture solution of ethanol and glycerin. Photographs were taken by an Olympus DP22 digital camera and processed with Adobe Photoshop CS6.

All specimens are deposited in the Institute of Karst Caves, Guizhou Normal University, Guizhou Province, China (**IKCGZNU**). The morphological terminology follows Qin et al. (2019).

## Taxonomy

### Tachycines (Gymnaeta) trapezialis sp. nov.

Taxon classificationAnimaliaOrthopteraRhaphidophoridae

EA277ADC-5190-5AA0-93F6-3394B3F07275

http://zoobank.org/00CE3ED0-336F-46C9-9BFD-AB3FB447CA6E

**Figure 1.**
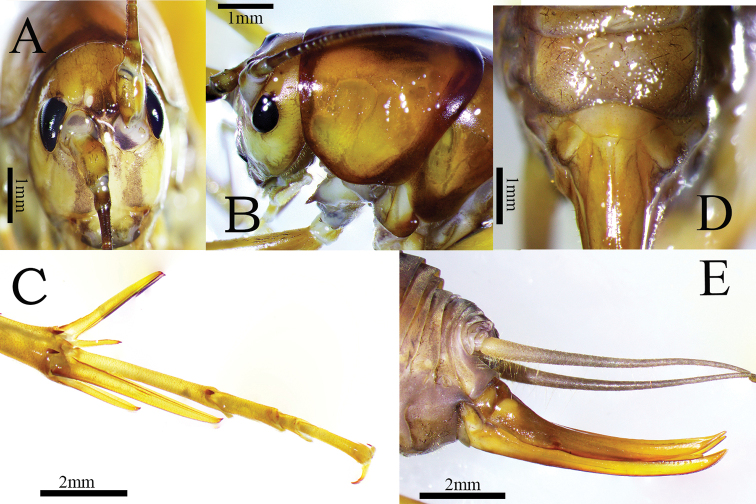
Tachycines (Gymnaeta) trapezialis sp. nov. **A** male; head and pronotum, dorsal view **B** male; head and pronotum, lateral view **C** male; hind tarsus in dorsal view **D** female, subgenital plate in ventral view **E** ovipositor in lateral view.

**Figure 2.**
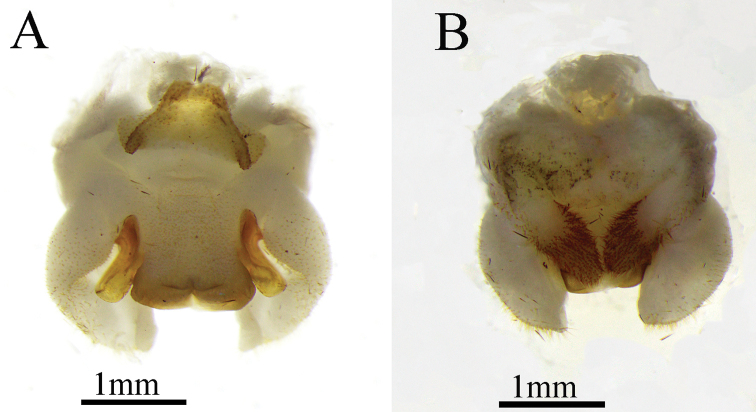
Tachycines (Gymnaeta) trapezialis sp. nov., male genitalia **A** dorsal view **B** ventral view.

**Figure 3.**
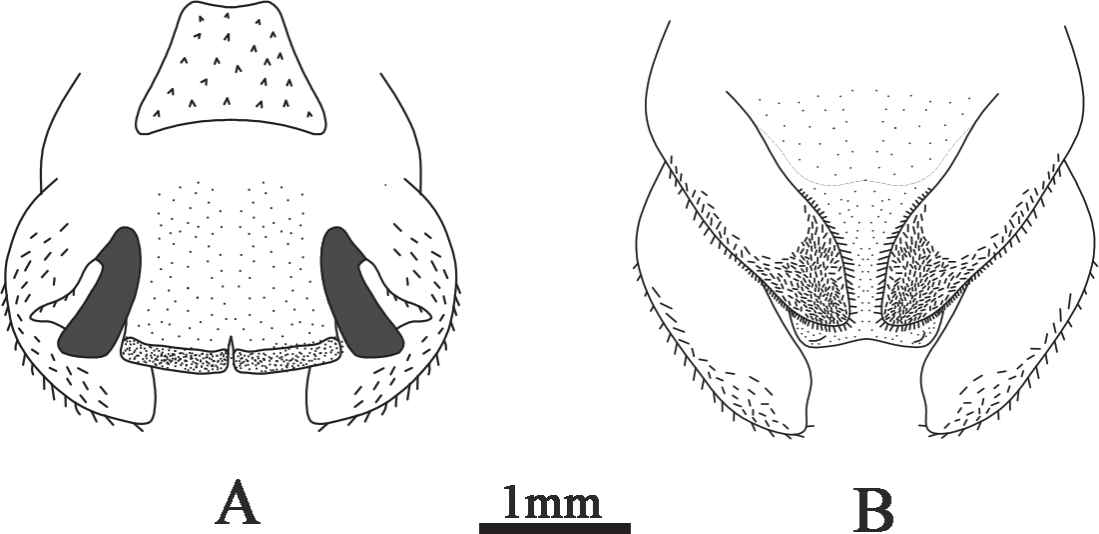
Illustration of male genitalia **A** dorsal view **B** ventral view.

**Figure 4.**
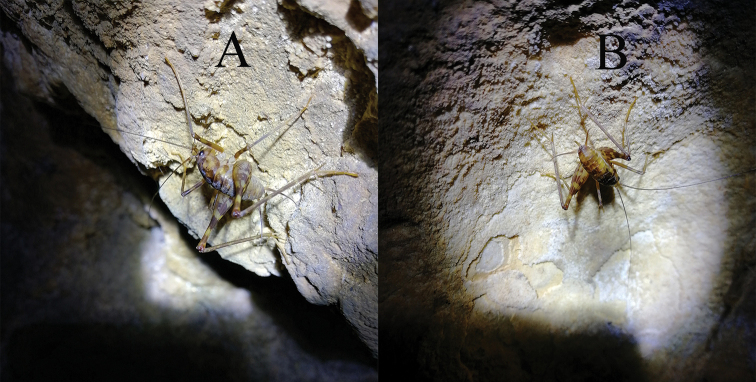
Tachycines (Gymnaeta) trapezialis sp. nov. nymphs from Diaosiyan Cave **A** male **B** female.

**Figure 5.**
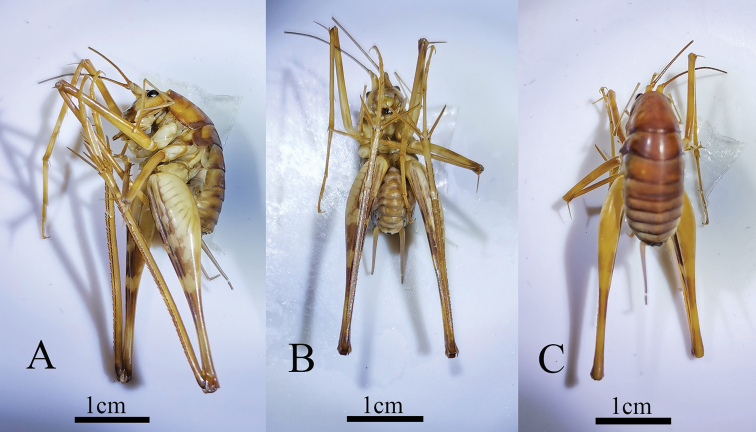
Holotype. Tachycines (Gymnaeta) trapezialis sp. nov. (male habitus) **A** lateral view **B** ventral view **C** dorsal view.

**Figure 6.**
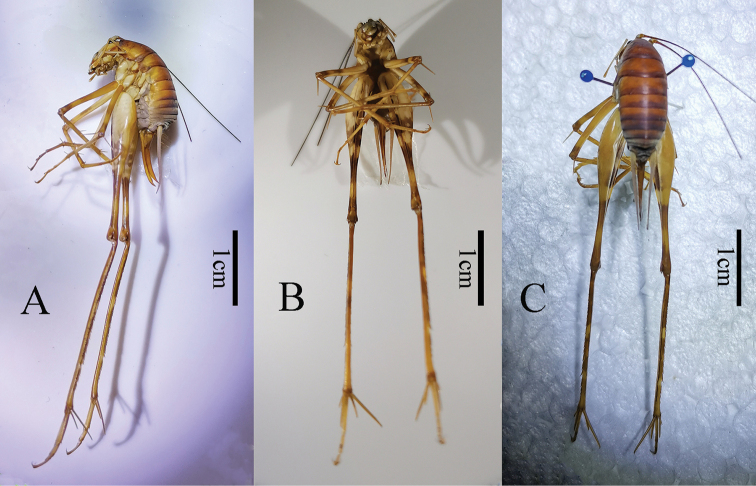
Tachycines (Gymnaeta) trapezialis sp. nov. (female habitus) **A** lateral view **B** ventral view **C** dorsal view.

Figures 1 , 2 , 3 , 4 , 5 , 6

#### Diagnosis.

This new species is very similar to T. (G.) lushuicus Qin, Liu & Li, 2019, T. (G.) parvus Qin, Liu & Li, 2019, and T. (G.) bifurcatus Gorochov, 2010, but differs from them in having the epiphallus of the male genitalia trapezoidal, without upper and lower deep notches and the hind tibia provided with 54–60 spines on each side for the new species. In T. (G.) lushuicus Qin, Liu & Li, 2019, the epiphallus of the male genitalia has an upper deep notch, and the hind tibia above has 61–67 spines on each side. In T. (G.) parvus Qin, Liu & Li, 2019, the epiphallus of the male genitalia has an upper and lower deep notch, and the female subgenital plate is triangular. In T. (G.) bifurcatus Gorochov, 2010, the epiphallus is strongly transverse, with a slightly notched upper part and medial projections on the lower part, and with a pair of large, almost oval lateral sclerites in males.

#### Type locality.

***Holotype***, 1♂, Diaosiyan Cave, Ziyun County, Guizhou, 25°35.06'N, 106°12.32'E, 1110–1120 m alt., October 2, 2019, collected by Xulin Zhou; ***paratypes***, 1♀, same data as holotype.

#### Specimens examined.

Diaosiyan Cave, Ziyun County, Guizhou Province: nymphs 11♂♂ 10♀♀, June 10, 2019, collected by Xulin Zhou, Juan Liao and Yi Du; 13♂♂ 9♀♀, October 2, 2019, collected by Xulin Zhou, Haixia Luo, Panpan Ren, Meizhen Deng and Suqin Zhao. Sanjiaoshan Cave, Ziyun County, Guizhou Province: 2♀♀, 25°35.35'N, 106°12.31'E, 1109m alt., October 2, 2019, collected by Xulin Zhou, Haixia Luo, Panpan Ren, Meizhen Deng and Suqin Zhao.

#### Description.

**Male.** Body medium-sized (Fig. 5). Vertex divided into two conical tubercles (Fig. 1A, B). Ommateum normal, not reduced; ocelli visible. Legs elongate and slender; fore femur about 1.6–1.8 times longer than the pronotum, ventrally unarmed, internal genicular lobe with a small spine; external genicular lobe with one elongate movable spur; ventral side of fore tibiae with two external spurs and two internal spurs. Mid femur ventrally unarmed, internal, and external genicular lobes with one elongate movable spur respectively; ventral side of mid tibiae with one external spur and one internal spur. Hind femur without spines ventrally; hind tibiae with 55–60 outer spines and 54–58 inner spines, arranged in groups. Supra-internal spurs of hind tibiae not exceeding ventral apex of hind tarsus (Fig. 1C). Hind tarsus keeled ventrally and with one dorsal apical spine. Male genitalia with trapezoidal epiphallus, lateral sclerites and median process divided at apical fourth (Figs 2A, B, 3A, B).

**Female.** Other characters are similar to male (Fig. 6). Subgenital plate wider than long and with three lobes; median lobe large and nearly trapezoid with apex transverse, paired lateral lobes small and nearly triangular with blunt apex (Fig. 1D). Ovipositor (Fig. 1E) shorter than half the length of hind femur.

#### Coloration.

Body brown. Frons with two dark longitudinal bands (Fig. 1A). Pronotum and mesonotum margins dark brown. Apexes of abdominal tergites dark brown. Hind femur with darkish stripes laterally.

#### Measurements (mm).

Body ♂16.2–17.6, ♀14.8–17.9; pronotum ♂6.3–6.6, ♀6.1–6.4; fore femur ♂10.8–11.3, ♀10.3–113; hind femur ♂16.3–17.6, ♀21.1–22.4; ovipositor 8.1–9.2.

#### Etymology.

The name refers to trapezoidal epiphallus in males.

#### Habitat.

**Figure 7.**
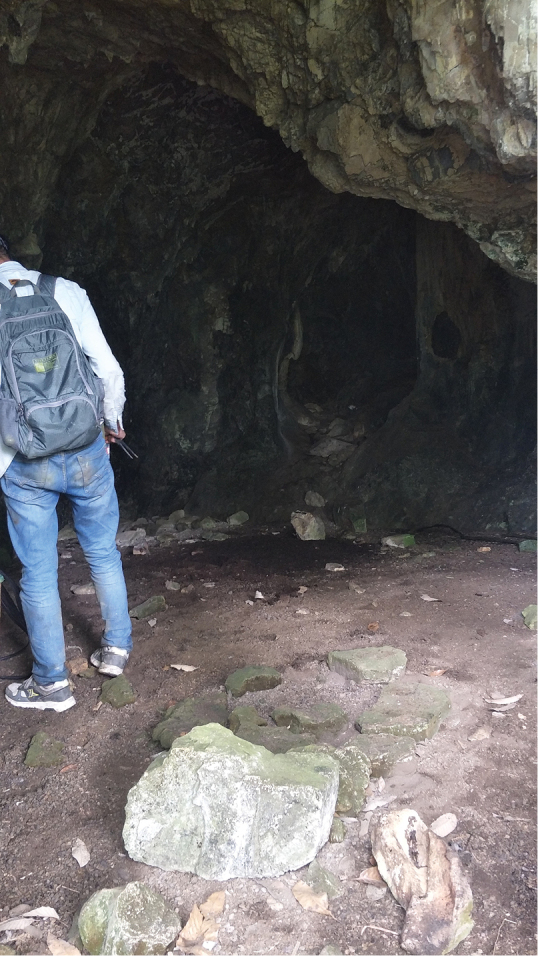
The entrance of Diaosiyan Cave (photographed by Yi Du from Luga village, Ziyun County, Guizhou).

Individuals of the new species live in groups in subtropical karst caves (Figs 4, 7).

#### Distribution.

Guizhou, China.

## Discussion

**Figure 8.**
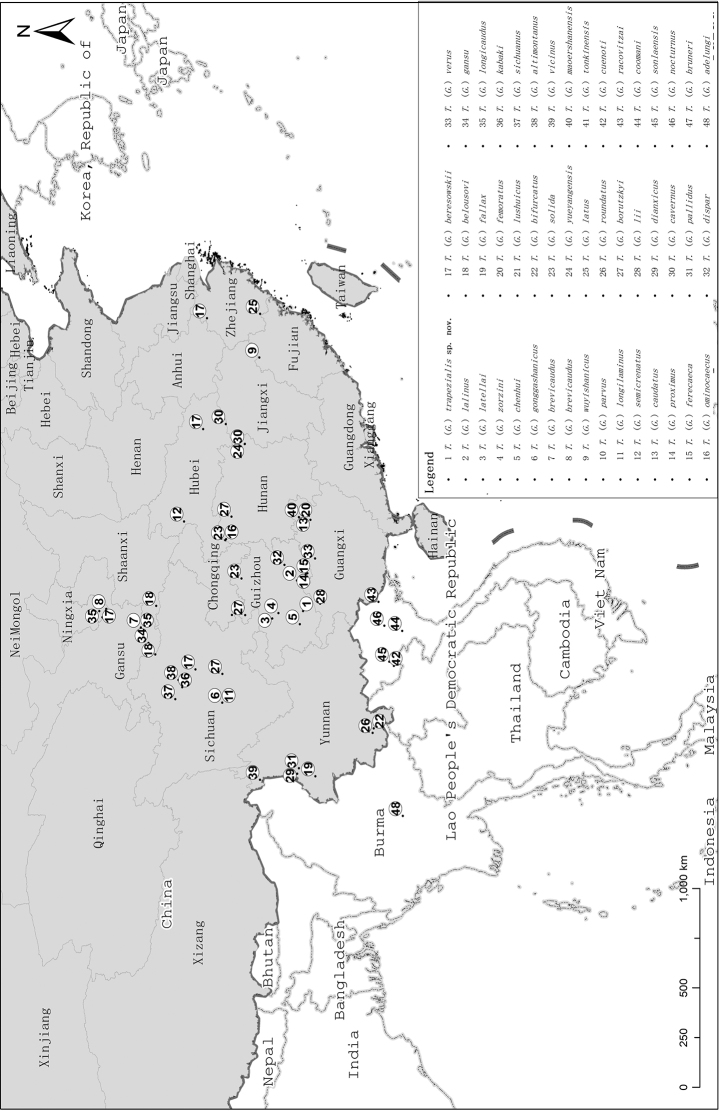
Distribution map for species of *Tachycines*, subgenusGymnaeta.

Species distribution of the subgenus (Fig. 8) presents a complexity which may reflect the degree of troglomorphism and parapatry distribution. Many species of this subgenus were found both inside and outside of cave. Eyes of these species vary from fully developed to reduced or absent, as in the totally blind T. (G.) omninocaecus. The geographical distribution pattern might be explained by the evolutionary scenario of zones of secondary admixture following epigean dispersal among lineages diverged from allopatry, as proposed by Ketmaier et al. (2013).

## Supplementary Material

XML Treatment for Tachycines (Gymnaeta) trapezialis
